# Evolution of Alignment and Clinical Outcomes During One Surgeon’s Learning Curve in L5-S1 Anterior Lumbar Interbody Fusion: A Single-Center Experience

**DOI:** 10.3390/jcm15051940

**Published:** 2026-03-04

**Authors:** Maxwell Sahhar, Manjot Singh, Derrick Kang, Jinseong Kim, Rhea D. Rasquinha, Joseph E. Nassar, Michael Farias, Zvipo Chisango, Nicolas Carayannopoulos, Todd Stafford, John Czerwein, Bassel G. Diebo, Alan H. Daniels

**Affiliations:** 1 Warren Alpert Medical School, Brown University, Providence, RI 02903, USA; 2 Department of Orthopedics, Brown University, Providence, RI 02903, USA

**Keywords:** ALIF, training, experience, quality improvement

## Abstract

**Background:** Anterior Lumbar Interbody and Fusion (ALIF) is particularly effective for improving radiographic alignment and functional outcomes. However, it also introduces distinct technical challenges, even for surgeons who are highly experienced with other lumbar fusion approaches. This study analyzes the effect of surgeon experience on clinical outcomes, radiographic parameters, and operative metrics in patients with degenerative lumbar disc disease undergoing single-level L5-S1 anterior lumbar interbody fusion. **Methods:** Adult patients who underwent L5-S1 ALIF with or without posterior fixation for degenerative disc disease between June 2017 and December 2024 were included. Patients were stratified into Early (from 2017 to December 2020), Middle (January 2021 to December 2022), and Recent (January 2023 to December 2024) groups. Demographics, radiographic alignment, in-hospital outcomes, and 2-year complication and reoperation rates were compared based on time of surgery. Multivariate logistic and linear regression adjusted for age, sex, BMI, comorbidities, prior fusion, and posterior instrumentation was conducted to assess the effect of accumulation of surgeon experience. **Results:** A total of 203 ALIFs were performed (mean age: 57.6 years; 50.7% female; mean Charlson Comorbidity Index: 2.1). Recent cases showed greater PT reduction (Early = 0.9°, Middle = −1.5°, Recent = −2.2°, *p* = 0.039), improved PI-LL mismatch correction (−0.4°, −4.8°, −5.4°, *p* = 0.007), higher L5-S1 lordotic correction (6.7°, 8.4°, 11.4°, *p* = 0.003), lower estimated blood loss (21.9 mL, 13.8 mL, 10.0 mL, *p* = 0.006), shorter OR time (107.4 min, 86.6 min, 75.2 min, *p* < 0.001), and fewer mechanical complications (39.3% vs. 13.7%, *p* < 0.001) and reoperations (10.7% vs. 2.1%, *p* = 0.023). Regression showed that each additional year of experience predicted improved alignment, lower blood loss and OR time, and reduced odds of complications (OR = 0.54, *p* < 0.001) and reoperations (OR = 0.49, *p* = 0.015). **Conclusions:** In this single-surgeon, single-center cohort, increasing ALIF-specific experience over time was associated with improvements in sagittal alignment, operative efficiency, and lower complication and reoperation rates. These findings describe the longitudinal learning curve of one surgeon and should be interpreted within this context.

## 1. Introduction

With career progression, spine surgeons accumulate clinical experience that refines patient selection, surgical planning, and operating room workflows. Previous studies have explored this, finding that greater experience and shorter operative time are associated with superior patient-reported outcomes [1,2,3]. High-volume spine surgeons, who prioritize efficiency and rapidly accumulate experience, demonstrate substantially lower complication rates and lower patient morbidity and mortality compared to their lower-volume counterparts [1,4]. Radiographic outcomes such as pelvic incidence-lumbar lordosis mismatch also show greater improvement when performed by more experienced surgeons [5]. This indicates that more experienced surgeons are more comfortable preparing endplates, utilizing cages with higher lordotic angles, and performing precise execution of alignment maneuvers [6,7].

However, for many techniques in which fellowship training already ensures proficiency, additional years of practice may not translate into measurable outcome differences. In a large study encompassing multiple techniques and indications, Muhly et al. reported that the only difference between patients treated by older versus younger spine surgeons was in patient satisfaction, with no measurable differences in clinical outcomes [8]. Similarly, in surgery for adolescent idiopathic scoliosis, experience was associated with improvements in intraoperative efficiency and postoperative pain, but not in complication rates [9]. These findings suggest that surgeon experience may have less impact on some procedures that are consistently performed during training. Importantly, posterior approaches are often the most common technique implemented during residency and fellowship training.

In the association between experience and outcomes, Anterior Lumbar Interbody Fusion (ALIF) can present a common and uniquely challenging learning curve. ALIF is particularly useful for restoring lordotic alignment at the lumbosacral junction and disc height [10,11,12,13]. Unlike posterior approaches, however, ALIF requires a true anterior exposure, vessel mobilization, and navigation of retroperitoneal anatomy, which are skills not developed through experience with posterior or lateral fusion techniques. Fellowship data also shows that trainees perform roughly half as many anterior cases as posterior cases overall, suggesting that surgeons enter independent practice with less anterior exposure experience [14]. Thus, even surgeons proficient in other fusion techniques may encounter a meaningful learning curve when incorporating ALIF into routine practice, diminishing the benefits to alignment observed in research environments. However, to date, no study has characterized the progression of this learning curve in surgeons implementing ALIF in their regular practice, especially with regard to alignment and complications.

From a clinical perspective, understanding how outcomes evolve with ALIF-specific experience has important implications for patient counseling, operative planning, and perioperative risk stratification. Surgeons early in their experience with ALIF may face distinct technical challenges that influence alignment correction and complication profiles, even when they are proficient with posterior approaches. Characterizing this progression allows for more informed expectations regarding operative efficiency and alignment outcomes during early adoption of the technique and may support the development of targeted training strategies and quality improvement initiatives aimed at mitigating early learning-curve-related risks. Therefore, this longitudinal study aims to characterize how outcomes, radiographic alignment, and intraoperative metrics evolve as ALIF is incorporated into regular practice. Importantly, this study is intended as a descriptive analysis of one surgeon’s longitudinal experience incorporating ALIF into practice, rather than a universal assessment of surgeon experience effects across institutions or surgeons.

## 2. Materials and Methods

### 2.1. Study Design

This was a retrospective single-center cohort study of patients seen by a single spine surgeon between 2017 and 2024 in the United States. Institutional review board approval was obtained prior to data collection and informed consent was acquired from each patient included in this study. This study was designed as a descriptive, single-surgeon, single-center analysis intended to characterize the longitudinal learning curve associated with the adoption of L5-S1 ALIF into independent practice rather than to draw generalized conclusions regarding surgeon experience across institutions.

The surgeon is a fellowship-trained spine surgeon who met all Accreditation Council for Graduate Medical Education (ACGME) case minimum requirements during training and began independent practice in 2013. Surgeon experience was operationalized as time in practice since initial licensure, rather than cumulative procedure count. Patients were stratified by calendar period (Early: 2017–2020; Middle: 2021–2022; Recent: 2023–2024), reflecting accumulating ALIF-specific experience over time. Although procedure volume contributes to technical proficiency, cumulative ALIF case counts prior to each surgery were not available for all patients and therefore could not be modeled directly.

Although the surgeon performed L5-S1 ALIF procedures prior to 2017, systematic collection of radiographic, perioperative, and complication data for this group of patients began in 2017. Consequently, earlier ALIF cases could not be included due to lack of accessible data. The present analysis therefore reflects outcomes from the period in which consistent data was available.

### 2.2. Patient Population

Patients were included in this study if they (i) were over the age of 18 years, (ii) underwent L5-S1 ALIF with or without posterior fixation for degenerative lumbar disc disease, and (iii) had radiographic and clinical outcomes available. Patients with a history of tumor, metastases, trauma, or infection were excluded. All included cases represented degenerative pathology; however, more granular diagnostic subtyping (e.g., discogenic pain, spondylolisthesis subtype) was not uniformly available and therefore could not be analyzed.

L5-S1 anterior lumbar interbody fusion was selected based on standard clinical decision-making including the need for restoration of disc height and segmental lordosis at the lumbosacral junction, patient-specific anatomy permitting safe anterior exposure, and surgeon judgment regarding the most appropriate fusion approach. When ALIF was not selected, alternative fusion techniques were used at the treating surgeon’s discretion. Outcomes of non-ALIF procedures were not evaluated in the present study. Radiographic measurements were performed retrospectively using standardized spinopelvic definitions; formal inter-rater reliability testing was not performed. All included cases consisted of standalone L5-S1 ALIF procedures without supplemental posterior fusion at the index operation. History of prior spinal surgery was recorded separately and adjusted for in multivariable analyses.

### 2.3. Data Extraction and Surgical Technique

Patient demographics, lumbo-pelvic alignment, and surgical complications were extracted from the study database. Demographic variables included age, sex, body mass index (BMI), Charlson Comorbidity Index (CCI), and history of spinal fusion. Lumbo-pelvic alignment parameters included pelvic tilt (PT), pelvic incidence (PI), pelvic incidence-lumbar lordosis (PI-LL) mismatch, L1-S1 lordosis, L4-S1 lordosis, and L5-S1 lordosis. In-hospital outcomes included estimated blood loss (EBL), operating room (OR) time, hospital length of stay (LOS), and rates of discharge to rehabilitation centers. Surgical complications included intraoperative great vessel injury, 90-day wound-related complications (i.e., superficial or deep wound infection, wound dehiscence), 2-year mechanical complications (i.e., cage subsidence, pseudoarthrosis, rod breakage, screw loosening, adjacent segment disease), and reoperations within 2 years of the index procedure.

Procedures were performed using a standard anterior approach to the L5-S1 disc space with the patient in the supine position. Interbody fusion was performed using commercially available ALIF cages. Detailed implant characteristics were not uniformly available and therefore were not analyzed separately.

Radiographic measurements were obtained using standard spinopelvic techniques on standing lateral radiographs. All radiographic measurements were made by trained research personnel using the Surgimap^®^ software (version 2.3, Audubon, PA, USA), a validated, reliable tool specifically for the spine. Repeat measurements were performed on a random subset to assess measurement consistency. Alignment parameters were extracted from the institutional database and verified for completeness prior to analysis. When multiple postoperative images were available, measurements were taken from the earliest postoperative standing radiograph available within the defined follow-up window.

Pelvic Tilt (PT) was measured as the angle between a vertical line originating at the center of the femoral heads and a line connecting the femoral head centers to the midpoint of the S1 superior endplate. Pelvic Incidence (PI) was calculated as the angle between a line perpendicular to the S1 superior endplate at its midpoint and a line connecting that point to the axis of the femoral heads. Lumbar Lordosis (LL), including global (L1-S1) and segmental (L4-S1 and L5-S1) parameters, was quantified using the Cobb angle between the superior endplate of the proximal vertebra and the superior endplate of S1. PI-LL Mismatch was determined by calculating the absolute difference between the measured pelvic incidence and the L1-S1 lumbar lordosis.

### 2.4. Statistical Analyses

Eligible patients were compared based on patient demographics, lumbo-pelvic alignment, and surgical complications using Student’s *t*-tests for continuous variables and chi-square tests for categorical variables. Multivariate logistic regression adjusting for age, sex, BMI, CCI, history of spinal fusion, and presence of posterior instrumentation was performed to evaluate the impact of surgeon experience on lumbo-pelvic alignment and surgical complications. Of note, Recent career patients were not analyzed for long-term postoperative complications since their 2-year outcome data were not yet available. All statistical analyses were conducted using SPSS Statistics for Windows, Version 29.0 (IBM Corporation, Armonk, NY, USA), with statistical significance defined as *p* < 0.05. Surgeon experience was treated as a continuous variable representing years since licensure at the time of surgery to model longitudinal changes in outcomes across the study period. Surgeon experience was modeled using calendar time since licensure rather than cumulative ALIF case volume, which was not uniformly available.

## 3. Results

### 3.1. Patient Characteristics

In total, 203 L5-S1 ALIF procedures were included, with 44 performed during the Early period (2017–2020), 88 during the Middle period (2021–2022), and 71 during the Recent period (2023–2024). The mean age was 57.6 years, 50.7% were female, and mean BMI was 29.12 kg/m^2^. The mean CCI was 2.11 and 24.1% reported a history of spinal fusion (Table 1). The mean preoperative pelvic tilt (PT) was 20.8 ± 9.5° and the mean pelvic incidence (PI) was 58.6 ± 13.5°. The mean PI-LL mismatch was 8.2 ± 15.6°. Regarding lumbar alignment, the mean lumbar lordosis (L1-S1) was −50.8 ± 15.3°, the mean L4-S1 lordosis was −32.4 ± 10.1°, and the mean L5-S1 segmental lordosis was 14.3 ± 8.2°. No patients underwent posterior fusion at the time of the index ALIF procedure. For patients who had prior spinal surgery, detailed diagnostic stratification and characterization of prior fusion constructs were not uniformly available and therefore could not be analyzed separately.

### 3.2. Spinopelvic Alignment

**Figure 1:**
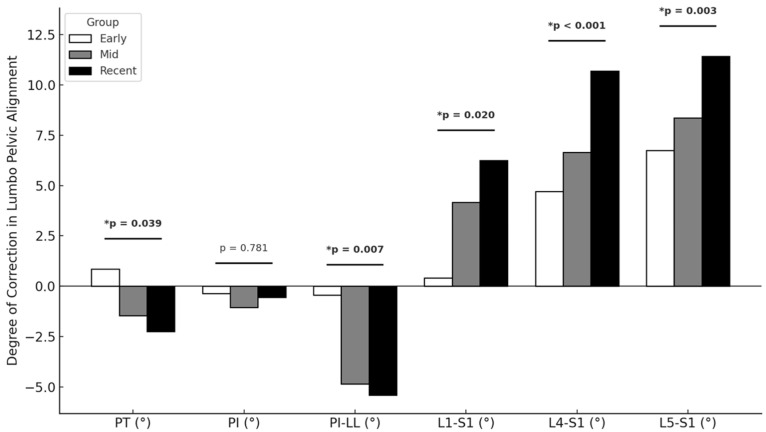
Improvement in Preoperative to 6-Week Postoperative Lumbo-Pelvic Alignment by Years of Surgeon Experience (* Statistically Significant).

By 6 weeks postoperatively, Recent Career cases showed larger reductions in PT (Early = 0.9°, Middle = 1.5°, Recent = −2.2°; *p* = 0.039) and PI-LL mismatch (−0.4°, −4.8°, −5.4°; *p* = 0.007), and larger improvements in L1-S1 (0.4°, 4.2°, 6.2°; *p* = 0.020), L4-S1 (4.7°, 6.6°, 10.7°; *p* < 0.001), and L5-S1 (6.7°, 8.4°, 11.4°; *p* = 0.003) lordosis. Multivariate regression analyses revealed that each additional year of surgeon experience was associated with a 0.8° decrease in ΔPT, 1.5° decrease in ΔPI-LL, 1.3° increase in ΔL1-S1, 1.5° increase in ΔL4-S1, and 1.4° increase in ΔL5-S1 (all *p* < 0.01) (Table 2, Figure 1).

### 3.3. In-Hospital Outcomes

Perioperatively, Recent Career cases had lower EBL (21.9 mL, 13.8 mL, 10.0 mL; *p* = 0.006) and OR time (107.4 min, 86.6 min, 75.2 min; *p* < 0.001), but higher rehab discharge rates (3.6%, 4.2%, 15.4%, *p* = 0.039). However, multivariate regression analyses revealed that only EBL (coeff = −3.9 mL) and OR time (coeff = −7.9 min) significantly decreased with each additional year of surgeon experience (all *p* < 0.001) (Table 3).

### 3.4. Surgical Complications and Reoperations

Two-year complication and reoperation analysis were limited to the Early and Middle cohorts, as patients in the Recent cohort had not yet reached a sufficient follow-up duration. By 2 years postoperatively, Middle Career cases noted fewer mechanical complications (39.3%, 13.7%, *p* < 0.001), specifically pseudoarthrosis (21.4%, 3.2%, *p* < 0.001) and adjacent segment disease (21.4%, 9.5%, *p* = 0.040). Furthermore, these cases also resulted in fewer reoperations (10.7%, 2.1%, *p* = 0.023). Multivariate regression analyses revealed that each additional year of surgeon experience was associated with 42% lower odds of wound-related complications, 46% lower odds of mechanical complications, specifically pseudoarthrosis (40% lower odds) and adjacent segment disease (32% lower odds), and 51% lower odds of reoperations (all *p* < 0.05) (Table 4). There were no recorded cases of intraoperative great vessel injury.

## 4. Discussion

This study examined associations of spinopelvic alignment, in-hospital outcomes, and complication rates with L5-S1 ALIF-specific operative experience. Importantly, these findings represent the longitudinal experience of a single surgeon at one institution and should be interpreted as a descriptive analysis of an individual learning curve rather than a universal assessment of surgeon experience effects in ALIF. With increasing years in the practice of ALIF, patients experienced greater restoration of segmental and global sagittal alignment, including larger corrections in PT, PI-LL mismatch, and lordosis at L1-S1, L4-S1, and L5-S1 levels. In-hospital metrics also improved, with lower EBL and shorter OR times in recent cases. Complication rates, including mechanical complications, pseudoarthrosis, and adjacent segment disease, were significantly lower in recent cases, and the odds of reoperation were halved. Taken together, this data supports the notion that the execution of ALIF benefits from a years-long learning curve of improvements in inherent skill, workflow refinement, and patient selection. Further, the potential alignment benefits achieved through ALIF may only be apparent with accumulated experience in the technique.

In terms of alignment, our findings agree with a variety of studies showing that spinopelvic correction generally improves with surgeon experience on a broad set of techniques [1,2,3]. Achieving optimal spinal alignment using ALIF is a complex task, requiring the integration of patient-specific anatomical parameters, intraoperative imaging feedback, surgical technique, and real-time judgment [4]. The accumulation of this surgical skill is reflected across multiple spinopelvic parameters commonly used to assess alignment [1,3]. Improved L5-S1 lordosis correction in surgeries performed later in the surgeon’s career indicates a growing ability to tailor segmental correction to patient-specific pelvic anatomy, thus contributing to more effective restoration of overall lumbar lordosis. The larger PI-LL correction observed in later-career cases further supports this trend. This suggests greater attention and ability to achieve a PI-LL mismatch under 10 degrees, an important metric for reducing risk of complications such as proximal junctional kyphosis, implant failure, and revision surgery [6]. Lastly, the larger pelvic tilt reduction suggests a greater awareness of global alignment goals and compensatory mechanisms in the lower extremities [4,5]. While these improvements in alignment correction may reflect improved technical execution and intraoperative judgment over time, it must also be noted that implant characteristics, cage lordotic angles, and disc preparation techniques were not directly measured and may reflect contemporaneous changes in available technology that contributed to improving alignment metrics over time [4,5].

In terms of in-hospital or intra-operative outcomes, our findings align with prior literature demonstrating improvements in operative time, EBL, and other indicators of operative efficiency over time in performing ALIF. Operative time generally decreases with greater experience in a technique due to a variety of interrelated factors. Ferguson et al. observed a significant decrease in operative time during the first two years of independent practice, suggesting a steep early learning curve for operative time attributable to skill and confidence [7]. However, these gains are not attributable to technical skill alone. In parallel, quality improvement initiatives, such as team-based sub-specialization, standardized intraoperative protocols, and streamlined workflows, have been shown to enhance operative efficiency [8,9,10,11]. These cumulative organizational changes are particularly impactful in early practice of particular exposures or techniques, when ongoing incremental refinement is critical to improving efficiency [11]. Shorter operative times have been shown to be a significant contributor to the improvements in EBL and other intra-operative outcomes investigated in the present study, in addition to improvements in confidence and skill [12]. Improvements in EBL are of the utmost importance given recent research indicating that a loss of greater than 2.3 L is clinically significant [13]. Improving EBL over the course of this surgeon’s career should be interpreted with caution, particularly in anterior spine surgery, where retroperitoneal blood pooling and difficulty distinguishing suctioned blood from irrigation fluid limit measurement precision. In this context, EBL is best understood as a surrogate marker of operative efficiency rather than an exact physiologic measure of hemorrhage. Reported estimated blood loss values likely underestimate true blood loss due to limitations inherent to anterior exposure and should therefore be interpreted comparatively across experience groups rather than as absolute measures. Of note, there was an increasing proportion of patients discharged to rehabilitation centers with increasing surgeon experience found by univariate analysis that was not found by multivariate analysis. Although univariate analysis demonstrated higher rates of discharge to rehabilitation in later cases, this association was not significant after multivariable adjustment. As such, increased rates of discharge should not be interpreted as a marker of worsening outcomes. Rather, this finding may reflect changes in patient complexity or institutional practices rather than surgeon experience alone.

Existing literature on complication rates in spine surgery similarly aligns with our findings, with several reports documenting a decrease in complications over time as surgeons gain experience, especially in technically demanding techniques [1,7,14,15,16,17,18,19]. Apart from intrinsic skill in ALIF, this again can be attributed to improved perioperative optimization [20,21]. In addition, the substantial correction in lordosis achieved may reduce mechanical stress on the adjacent segments, thus decreasing the rates of adjacent level degeneration and implant failure [22]. It follows that rates of reoperation are lower as a result. Although greater lordosis restoration may plausibly influence mechanical complication risk [23], the present study was not designed to evaluate causal relationships between the magnitude of alignment correction and specific complications. We therefore interpret these proposed mechanisms as hypothesis-generating rather than directly tested in this cohort. Future studies incorporating planned correlation analyses, as well as longer-term radiographic follow-up, are needed to determine whether the degree of lordosis correction independently predicts mechanical complications such as pseudoarthrosis or adjacent segment disease.

The findings of this study should be interpreted cautiously due to limitations inherent to the single-center, single-surgeon design. Although this structure enables a longitudinal assessment of one surgeon’s experience, it limits generalizability and precludes separation of surgeon-specific factors from broader temporal changes in practice. Observed improvements over time may therefore reflect not only increasing technical proficiency with ALIF, but also concurrent evolution in implant technology, perioperative protocols, imaging, anesthesia management, and postoperative care pathways. Additionally, patient selection criteria for ALIF may have changed over time to favor more complex cases as surgeon experience increased, introducing potential selection bias that cannot be fully accounted for in retrospective analyses.

Alternative explanations for the observed trends must therefore be considered. Improvements in operative time and estimated blood loss may reflect refinements in operating room workflow, team familiarity, and institutional efficiency initiatives in addition to individual technical mastery. Similarly, alignment gains and reductions in mechanical complications may be influenced by evolving implant designs or surgical adjuncts that were not uniformly captured in the present dataset. These factors cannot be isolated from surgeon experience in this study and should be considered when interpreting the results

Despite these limitations, the findings of this study align with previously published literature demonstrating learning-curve effects across many complex procedures, including deformity correction and osteotomy techniques, where complication rates and operative efficiency improve with experience [24,25]. However, literature specifically characterizing learning curves in ALIF remains limited. As ALIF involves distinct anterior exposure and other considerations that differ from posterior approaches, longitudinal single-surgeon analyses may provide valuable insight into progression in technique for spine surgeons. Consequently, these findings may inform training efforts for surgeons transitioning to anterior approaches. Furthermore, identifying this longitudinal trajectory allows for the implementation of standardized perioperative protocols designed to mitigate risks during a surgeon’s early experience with the technique. Ultimately, recognizing ALIF as a procedure with a prolonged learning curve emphasizes the requirement for continuous outcome tracking and iterative workflow refinement to ensure the delivery of high-value, durable spinal care.

### Limitations

This study has several important limitations. First, it reflects the experience of a single surgeon at a single institution, limiting generalizability and precluding separation of surgeon experience from individual practice style. Second, surgeon experience was modeled using calendar time rather than cumulative case volume and evolving implant technology, hospital protocols, and perioperative practices over the seven-year period may have contributed to the observed trends. Third, patient selection criteria for ALIF may have changed over time, introducing selection bias. Fourth, patient-reported outcomes were not available for this cohort, limiting assessment of functional improvement. Fifth, radiographic outcomes were assessed only at short-term follow-up, and long-term durability of alignment correction was not evaluated. Sixth, two-year complication and reoperation data were unavailable for the most recent cohort, restricting longitudinal comparisons. Seventh, the absence of outcome data for L5-S1 ALIF procedures prior to 2017 limits the characterization of the earliest phase of the surgeon’s learning curve and precludes comparison of later outcomes to very early outcomes. However, the seven-year time period described captures substantial longitudinal experience during which meaningful changes in technique, workflow, and outcomes were achieved. Eighth, first assistant involvement varied throughout the study period as procedures were performed in an academic setting with rotating residents and fellows; the degree of assistant experience was not captured and may represent an unmeasured confounder. Finally, the absence of a comparator surgeon or alternative surgical approach limits causal inference.

## 5. Conclusions

In this investigation, increased surgeon experience in ALIF, as measured by time in practice, was associated with improved spinopelvic alignment, improved in-hospital metrics, and reduced complication and reoperation rates following L5-S1 ALIF. In this single-surgeon, single-center study, increasing ALIF-specific experience over time was associated with improvements in alignment, operative metrics, and complication rates. These results characterize one surgeon’s learning curve and highlight how technical mastery, workflow refinement, and patient selection may evolve during adoption of ALIF. The findings should not be generalized to all surgeons or practice settings but may inform expectations during early experience with this technique. These findings suggest that longitudinal surgical experience contributes meaningfully to patient selection, operative workflow, technical execution, and intraoperative decision-making in ALIF, all of which collectively contribute to safer and more effective outcomes over time.

## Figures and Tables

**Table 1:** Patient Characteristics.

	Overall	Early (2017–2020)	Middle (2021–2022)	Recent (2023–2024)
No. of Patients	203	44	88	71
Age (Mean ± SD)	57.6 ± 12.5	52.0 ± 12.0	58.0 ± 12.9	60.5 ± 11.1
Gender (Female)	103 (50.7%)	26 (59.1%)	43 (48.9%)	34 (47.9%)
BMI (Mean ± SD)	29.1 ± 5.4	28.8 ± 5.6	29.0 ± 5.7	29.5 ± 4.9
CCI (Mean ± SD)	2.1 ± 1.7	1.4 ± 1.4	2.3 ± 1.9	2.4 ± 1.6
Osteoporosis	11 (6.0%)	1 (2.5%)	7 (9.1%)	3 (4.5%)
Prior Fusion	49 (24.1%)	12 (27.3%)	20 (22.7%)	17 (23.9%)
PT	20.8 ± 9.5	16.3 ± 9.4	21.6 ± 9.2	22.4 ± 9.3
PI	58.6 ± 13.5	56.1 ± 15.5	59.5 ± 13.6	58.8 ± 12.0
PI-LL	8.2 ± 15.6	1.5 ± 12.2	8.9 ± 15.1	10.9 ± 17.0
L1-S1	−50.8 ± 15.3	−54.1 ± 16.4	−50.9 ± 15.1	−48.6 ± 14.8
L4-S1	−32.4 ± 10.1	−34.6 ± 11.0	−33.6 ± 9.9	−29.5 ± 9.2

**Table 2:** Preoperative to 6-Week Postoperative Improvement in Lumbo-pelvic Alignment.

	*T*-Test				Multivariable Regression		
Parameter	Early (N = 56)	Mid (N = 95)	Recent (N = 52)	*p*-Value	B	95% CI	*p*-Value
PT (°)	0.85 (6.31)	−1.46 (6.88)	−2.24 (5.55)	**0.039**	−0.83	−1.46 to −0.20	**0.010**
PI (°)	−0.35 (5.95)	−1.04 (6.29)	−0.54 (5.22)	0.781	−0.30	−0.90 to 0.31	0.335
PI-LL (°)	−0.44 (7.98)	−4.84 (10.03)	−5.39 (7.46)	**0.007**	−1.48	−2.29 to −0.68	**<0.001**
L1-S1 (°)	0.41 (8.34)	4.17 (10.10)	6.24 (8.33)	**0.020**	1.33	0.35 to 2.30	**0.008**
L4-S1 (°)	4.70 (6.89)	6.64 (8.00)	10.68 (5.77)	**<0.001**	1.48	0.81 to 2.16	**<0.001**
L5-S1 (°)	6.74 (6.58)	8.36 (7.97)	11.41 (5.09)	**0.003**	1.42	0.75 to 2.08	**<0.001**

Significant values are bolded.

**Table 3:** In-Hospital and Out-Of-Hospital Outcomes.

	*T*-Test				Multivariable Regression		
Variable	Early (N = 56)	Mid (N = 95)	Recent (N = 52)	*p*-Value	B/OR	95% CI	*p*-Value
EBL	21.86 (26.06)	13.75 (19.48)	10.00 (0.00)	**0.006**	−3.89	−5.67 to −2.11	**<0.001**
OR Time	107.39 (26.65)	86.62 (33.26)	75.20 (17.35)	**<0.001**	−7.87	−10.41 to −5.34	**<0.001**
LOS	2.82 (1.65)	3.38 (2.60)	3.71 (2.11)	0.131	0.16	−0.04 to 0.36	0.114
Rehab Discharge	2 (3.6)	4 (4.2)	8 (15.4)	**0.039**	1.38	0.78 to 2.44	0.267

Significant values are bolded.

**Table 4:** Postoperative Complications (Early vs. Middle Cohorts Only).

	Chi-Squared Test			Multivariable Regression		
Complication	Early (N = 56)	Mid (N = 95)	*p*-Value	OR	95% CI	*p*-Value
Wound-Related	6 (10.7)	4 (4.2)	0.121	0.58	0.33 to 1.00	**0.050**
Mechanical	22 (39.3)	13 (13.7)	**<0.001**	0.54	0.39 to 0.75	**<0.001**
Cage Subsidence	4 (7.1)	3 (3.2)	0.261	0.61	0.31 to 1.20	0.151
Pseudoarthrosis	12 (21.4)	3 (3.2)	**<0.001**	0.60	0.40 to 0.88	**0.010**
Rod Breakage	1 (1.8)	1 (1.1)	0.703	0.31	0.02 to 4.04	0.369
Screw Loosening	1 (1.8)	3 (3.2)	0.612	1.38	0.58 to 3.30	0.465
Adjacent Segment Disease	12 (21.4)	9 (9.5)	**0.040**	0.68	0.47 to 0.98	**0.037**
Reoperation	6 (10.7)	2 (2.1)	**0.023**	0.49	0.27 to 0.87	**0.015**

Significant values are bolded.

## Data Availability

The data presented in this study are not publicly available due to patient privacy and protected health information restrictions.
